# Serum brain-derived neurotrophic factor levels and personality traits in patients with major depression

**DOI:** 10.1186/s12888-015-0413-1

**Published:** 2015-03-04

**Authors:** Hiroshi Nomoto, Hajime Baba, Emi Satomura, Hitoshi Maeshima, Naoko Takebayashi, Yuki Namekawa, Toshihito Suzuki, Heii Arai

**Affiliations:** 1 Department of Psychiatry, Juntendo University, School of Medicine, 2-1-1 Hongo, Tokyo, Bunkyo-ku Japan; 2 Juntendo University Mood Disorder Project (JUMP), Department of Psychiatry, Juntendo University Koshigaya Hospital, 560 Fukuroyama, 343-0032 Koshigaya, Saitama Japan

**Keywords:** Depression, BDNF, Personality, Self-directedness, TCI

## Abstract

**Background:**

Brain-derived neurotrophic factor (BDNF) is a member of the neurotrophin family of growth factors. Previous studies have demonstrated lower serum BDNF levels in patients with major depressive disorder (MDD) and reported an association between BDNF levels and depression-related personality traits in healthy subjects. The aim of the present study was to explore for a possible association between peripheral BDNF levels and personality traits in patients with MDD.

**Methods:**

In this cross-sectional study, a total of 123 inpatients with MDD (*Diagnostic and Statistical Manual for Mental Disorders*, 4th edition) at the Juntendo University Koshigaya Hospital were recruited. Serum levels of BDNF were measured. Personality traits were assessed using the 125-item short version of the Temperament and Character Inventory (TCI).

**Results:**

Multiple regression analysis adjusted for age, sex, body mass index, dose of antidepressant, and depression severity showed that TCI Self-Directedness (SD) scores were negatively associated with serum BDNF levels (β = −0.23, p = 0.026).

**Conclusions:**

MDD patients who have low SD did not show the reduction in serum BDNF levels that is normally associated with depressive state. Our findings suggest that depression-related biological changes may not occur in these individuals.

## Background

Brain-derived neurotrophic factor (BDNF) is a member of the neurotrophin family of growth factors, which also includes nerve growth factor, neurotrophin-3, and neurotrophin-4/5 [1]. BDNF is the most abundant neurotrophin in the brain, playing a critical role in growth, differentiation, maintenance, and synaptic plasticity. Animal models demonstrate a stress-induced dysregulation of BDNF expression, especially in response to chronic stress [2]. In humans, lower serum BDNF levels have been found in patients with major depressive disorder (MDD) [3,4], bipolar disorder [5,6], schizophrenia [7,8], eating disorders [9,10], obsessive-compulsive disorder [11], and alcohol dependence [12]. In most studies involving MDD, serum BDNF levels have been shown to correlate negatively with disease severity [13-15], and antidepressant treatment increases serum levels of BDNF [14,16-19]. However, there is a large overlap between BDNF levels in the serum of depressed patients and controls [15], and levels are not decreased in all depressed patients.

The Temperament and Character Inventory (TCI) is based on a psychobiological model that describes the structure and development of personality with four temperament and three character dimensions. In this psychobiological model, Cloninger et al. [20] postulated that temperament is genetically independent, emerges in early life, and influences subsequent perceptual memory and habit formation. In addition, they propose that character is determined by learning about self-concepts, matures in adulthood, and affects personal and social lives over time. The temperament dimensions of the TCI consist of Novelty-Seeking (NS), Harm-Avoidance (HA), Reward-Dependence (RD), and Persistence (P). The three character dimensions are Self-Directedness (SD), Cooperativeness (C), and Self-Transcendence (ST). A number of studies using the TCI suggest that depressed patients show high HA and low SD, and these personality traits are associated with the state of depression [21-28]. Hansenne et al. [24] administered the TCI to 40 patients with MDD and 40 healthy controls. Depressed patients exhibited higher HA and ST scores as well as lower SD and C scores compared to controls. The three other dimensions did not differ between depressed patients and controls. Among the depressed group, HA, SD and C dimensions were related to the severity of depression as assessed by the Hamilton Rating Scale for Depression (HAM-D). Kimura et al. [21] measured personality traits in 131 remitted patients with MDD and 154 normal controls. Patients with MDD had significantly higher HA scores and significantly lower SD and C scores. Hirano et al. [27] evaluated 108 patients with MDD using the TCI before and after a 16-week antidepressant treatment. The level of depression, as assessed by HAM-D, was correlated positively with the HA score and negatively with the SD and C scores. Moreover, treatment both reduced the severity of depression and normalized scores on these three dimensions. More recently, Celikel et al. [26] used the TCI in 81 outpatients (67 women, 14 men) and 51 healthy controls (35 women, 16 men). Depressed patients exhibited significantly higher HA scores and lower SD scores compared with healthy controls. Hur et al. [28] also examined 56 patients with MDD and the same number of age-, sex-, and education-matched normal controls using the TCI. MDD patients had significantly higher HA scores and significantly lower scores for SD and C and subscales of NS, RD, and ST than normal controls. Kaneda et al. [25] measured TCI in 93 patients with MDD before and after 6 weeks of selective serotonin re-uptake inhibitor (SSRI) treatment. Compared with 91 normal control participants, patients with MDD had less NS and SD and greater HA. Moreover, they found early treatment responders showed less HA and more SD than late responders. Kampman et al. [23] measured traits using the TCI in 98 patients with MDD before and after 6 weeks of SSRI treatment. MDD patients had significantly higher HA scores compared with community samples both at baseline and endpoint, and HA was the trait that accounted for the most variability in the post-treatment Montgomery Asberg Depression Rating Scale (MADRS) scores. The authors concluded that HA was associated with the risk of and treatment response to depression. A recent systematic review and meta-analysis [22] demonstrated that high HA was associated with current depressive symptoms and with depressive trait. These previous reports suggest that HA and SD scores with depression are more consistently associated with depression than other dimensions of TCI. The HA score quantifies the extent to which a person is anxious, pessimistic, and shy or risk-taking, optimistic, and outgoing. The SD score quantifies executive functions, such as responsibleness, purposefulness, and resourcefulness. A high HA score indicates that a person is anxiety prone, which may create an emotional venerability to depression [29]. A high SD score indicates that an individual may lack executive functions that protect a person from depression, and a low SD score indicates immature personality [29].

Previous reports have only examined the relationships between blood BDNF levels and personality traits in healthy subjects [30-34]. These studies demonstrated an association between peripheral BDNF levels and depression-related personality traits. To our knowledge, however, no study has examined the relationship between peripheral BDNF levels and personality traits in patients with MDD. While serum BDNF levels are negatively correlated with depressive symptoms, not all depressed patients display lower serum BDNF levels than controls [15]. We hypothesize that serum BDNF levels do not decrease in MDD patients with high HA or low SD because these traits may enhance susceptibility to depression even in the absence of biological changes. The aim of the present study was to explore the possible association between peripheral BDNF levels and personality traits evaluated by the TCI in patients with MDD. Thus, we analyzed the association between HA and SD scores and serum BDNF levels. This study is a part of the Juntendo University Mood Disorder Project (JUMP).

## Methods

### Subjects

In this cross-sectional study, a total of 123 inpatients with who met the *Diagnostic and Statistical Manual for Mental Disorders*, 4th edition (DSM-IV) criteria for MDD were recruited from the Juntendo Koshigaya Hospital between May 2006 and December 2013. The diagnosis was done by senior psychiatrists. Exclusion criteria included history of other psychiatric disorders including delusions, severe or acute medical illnesses, neurological disorders, use of drugs that may cause depression, clinical evidence of dementia, and Mini-Mental State Examination scores <24. Psychotic symptoms were present in 19 patients. Twenty-five patients were smokers. With regard to chronic physical problems, 20 patients had hypertension (HT), 11 patients had hyperlipidemia (HL), and eight patients had diabetes mellitus (DM). Depressive symptoms were assessed using the HAM-D [35] on admission to the hospital. All patients were on antidepressant medication but were not responding to their current medication. The antidepressant doses were converted to equivalent doses of imipramine [36]. Numbers of depressive episodes were confirmed via medical records.

All study protocols were approved by the Medical Ethics Committee of Juntendo University Koshigaya Hospital and were performed in accordance with the regulations outlined by Juntendo University. All participants provided written informed consent prior to participation.

### Assessment of personality traits

Personality traits were assessed using the 125-item short version of TCI [37]. Reliability and validity of the Japanese version in a nonclinical population [37] and factor validity in outpatients with major depression [38] have been reported.

The temperament dimensions of the TCI measure individual differences in emotional responses to associatively conditioned stimuli. The four temperaments are HA (i.e., anxious versus risk-taking), NS (i.e., impulsive versus rigid), RD (i.e., approval seeking versus aloof), and P (i.e., overachieving versus underachieving). The character dimensions measure individual differences in higher cognitive processes that modulate emotional conflicts to satisfy a person’s goals and values. The character dimensions quantify the three branches of mental self-government: SD (executive functions, such as being responsible, purposeful, and resourceful), C (legislative functions, such as being tolerant, forgiving, and helpful), and ST (judicial functions, such as being intuitive, judicious, and aware) [29].

### Serum BDNF measurements

Blood samples were taken at 07:00, before breakfast, on the day after admission and were centrifuged immediately after blood was drawn and clotting confirmed. Serum samples were stored at -80°C until the time of processing. A Quantikine Human BDNF Immunoassay Kit (R&D Systems, Minneapolis, MN, USA) was used according to the instructions from the manufacturer and as previously described [33]. The detection limit was 20 pg/ml and the intra-assay coefficient of variation was <8%.

### Statistical analysis

Correlations between serum BDNF levels, HA or SD scores, and possible confounding factors (age, sex, daily dose of antidepressants, HAM-D total score, presence of psychotic symptoms, age of onset, education, smoking, presence of HT, HL, DM, and body mass index [BMI]) were analyzed using Spearman’s rank correlation coefficient. Multiple regression analysis was conducted using serum BDNF levels as a dependent variable and confounding factors, HA score, and SD score as independent variables. The significance level was p < 0.05. Statistical procedures were performed using the Japanese version of SPSS v15.1 (SPSS Inc., Japan, Tokyo, Japan).

## Results

Participants (N = 123) included 44 men and 79 women with a mean age of 56.8 years (range, 31–85 years). Participant characteristics are shown in Table 1. Serum BDNF levels were negatively correlated with age (R = −0.38, p < 0.001) and age of onset (R = −0.26, p = 0.003) and positively correlated with sex (R = 0.24, p = 0.009), daily dose of antidepressants (R = 0.26, p = 0.008), and BMI (R = 0.30, p = 0.001). Correlations between serum BDNF levels and other possible confounding factors were not significant. HA score was negatively correlated with age (R = −0.24, p = 0.008) and age of onset (R = −0.18, p = 0.04) and was not correlate with other possible confounding factors. No correlations between SD score and confounding factors were significant. There was a strong, positive correlation between age of onset and age. From these results, we assumed age, sex, BMI, daily dose of antidepressants, and HAM-D score as significant confounding factors.

**Table 1:** **Characteristics of study participants**

	Patients with MDD
	(n = 123)
	Mean ± SD
Age (years old)	56.8 ± 14.1
Sex (F/M)	79/44
Education (years)	13.0 ± 2.6
BMI (%)	23.0 ± 3.5
Age of onset (years old)	49.9 ± 15.8
HAM-D total score	22.0 ± 9.8
Number of depressive episodes	2.0 ± 1.2
Duration of current episode (months)	11.9 ± 13.2
Daily dose of antidepressant (mg)^a^	129.5 ± 98.3
Novelty-Seeking (NS)	7.7 ± 2.8
Harm-Avoidance (HA)	13.9 ± 4.1
Reward-Dependence (RD)	9.2 ± 2.5
Persistence (P)	2.2 ± 1.6
Self-Directedness (SD)	14.6 ± 5.1
Cooperativeness (C)	16.6 ± 3.4
Self-Transcendence (ST)	3.9 ± 2.9
Serum BDNF level (pg/ml)	19283.9 ± 7952.6

^a^Antidepressants were converted into equivalent doses of imipramine.

MDD, major depressive disorder; SD, standard deviation; F, female; M, male; BMI, body mass index; HAM-D, Hamilton Rating Scale for Depression; BDNF, brain-derived neurotrophic factor.

Multiple regression analysis showed that age (β = −0.38, p < 0.001) and SD score (β = −0.23, p = 0.026) were significantly associated with serum BDNF levels after controlling for HAM-D total score and other confounding factors. Sex, HAM-D total score at admission, daily dose of antidepressants, BMI, and HA score were not significantly associated with serum BDNF levels (Table 2).

**Table 2:** **Results of multiple regression analysis of serum BDNF levels**

	β	p-value
Age	-0.38	<0.001
Sex	-0.07	0.526
HAM-D total score at admission	0.03	0.774
Daily dose of antidepressant	0.12	0.199
BMI	0.08	0.429
Harm-Avoidance (HA)	-0.05	0.613
Self-Directedness (SD)	-0.23	0.026

BDNF, brain-derived neurotrophic factor; HAM-D, Hamilton Rating Scale for Depression, BMI, body mass index.

## Discussion

The present study demonstrated that there was an inverse relationship between serum BDNF levels and scores for the SD personality trait in patients with MDD.

In terms of the association between peripheral BDNF levels and personality traits, Lang et al. [30] reported a negative correlation between serum BDNF levels and neuroticism in 118 healthy subjects, evaluated by the Neuroticism-Extroversion-Openness (NEO) Five-Factor Inventory. Terracciano et al. [39] reported that serum BDNF levels were inversely related to neuroticism, measured by the Revised NEO Personality Inventory in a large community-based study (n = 2099). This association of serum BDNF levels with neuroticism was independent of depressive symptoms. Okuno et al. [32] reported a positive correlation between plasma BDNF levels and the extroversion score measured by the NEO Five-Factor Inventory in 269 healthy employees. Other studies have evaluated personality traits using the TCI. Minelli et al. [33] reported a negative correlation between HA scores and serum BDNF levels in 107 healthy subjects. Yasui-Furukori et al. [34] also reported that plasma BDNF levels were negatively correlated with HA scores and positively correlated with SD scores in 178 healthy subjects. Inconsistent with results of these previous studies [33,34], we found that serum BDNF levels were not correlated with HA scores and were negatively correlated with SD scores in patients with MDD. However, these previous studies were performed in healthy subjects. To our knowledge, this article is the first to report an association between serum BDNF levels and personality traits in patients with MDD. The discrepancy in these results may be caused by the differences in diagnosis of subjects.

Previous studies have demonstrated lower serum BDNF levels in depressed patients than in controls [13,16,40]. Similarly, we previously found lower serum BDNF levels in 109 patients with MDD compared with 163 healthy controls [15]. From these previous studies, serum BDNF levels may reflect a depression-related biological change. However, we found a large overlap in patients with MDD and controls (mean levels and standard deviations were 20,321.2 ± 8180.0 pg/ml in MDD patients and 27,105.5 ± 8310.2 pg/ml in controls), and there were patients with MDD whose serum BDNF levels were not lower than the control mean. In the present study, we hypothesized that the variability in serum BDNF levels was due to an absence of depression-related biological changes in MDD patients with weak SD, and this hypothesis was supported by the negative correlation between serum BDNF and SD. Previous studies suggest that SD is a marker of executive functions that protect a person from depression, and low SD scores indicate immaturity [29]. Individuals with low SD may be more likely to fall into a depressive state when experiencing undesirable, though not overtly stressful, situations in daily lives. Thus, biological changes caused by strong and continuous stress may be less likely to occur in MDD patients with low SD personality trait. In summary, the depressive state in MDD patients with low SD personality trait may be affected more by psychological than biological factors.

A key limitation of the present study was that all patients with MDD were on antidepressant medications. However, serum BDNF levels were not influenced by the type (data not shown) or dose (Table 2) of antidepressant. Yoshimura et al. [19] found that paroxetine and milnacipran increased serum BDNF levels to similar levels in responders. Serum BDNF levels may change only with successful medication [19], and our patients were not responsive to the medications they were receiving at the time of the study. Although we did not find a significant association between antidepressants and BDNF levels, follow-up studies in drug-naïve patients should be conducted. Genetic considerations were beyond the scope of this study, but future work should explore the VAL/MET polymorphism of the BDNF gene as a biomarker. Additional limitations of this study include small sample size and an exclusion of other personality traits besides SD and HA. Future studies should explore a wider array of personality traits using larger samples.

## Conclusions

Serum BDNF levels are negatively associated with the personality trait of SD in patients with MDD. While most patients with MDD have reduced serum BDNF levels, the patients with lower SD showed higher serum BDNF levels than other patients with MDD, suggesting that a depressive state is not linked with depression-related biological changes in these patients.
